# The Principles and Practice of Obstetrics

**Published:** 1861-11

**Authors:** 


ART. VII.—*The Principles and Practice of Obstetrics.* By GUNNING S. BEDFORD, A.M., M.D., Professor of Obstetrics, etc. in the University of New York. Illustrated by Four Colored Lithographic Plates and Ninety-nine Wood Engravings. 8vo., pp. 731. New York: S. & W. Wood, 1861.

THE one cardina object which the author states he has had constantly before him—to be useful—has certainly been attained in the handsome volume which forms the subject of this notice. We regret that we cannot give a more extended review of its contents, but we have examined the work sufficiently to warrant us in expressing the conviction that both in its matter and arrangement it will be very acceptable to the general practitioner as well as to teachers of obstetrics. There are few points left untouched, and the skillful obstetrician wields a ready pen on every page. It is a complete treatise on the subject which it discusses, and is very full upon matters which are but lightly dwelt upon in many of the treatises on obstetrics.

We cannot, of course, in this short notice, discuss with the author theories and principles, but we can speak with commendation of the thorough and successful investigation by him of the difficult points of midwifery, for which the practitioner will feel especially grateful. The volume is issued in a superior manner, creditable alike to author and publishers; though, so far as the latter are concerned, we confess to a much higher admiration for the beautifully colored lithographic plates than for the indifferently executed wood-cuts.

